# Do We Preserve Tumor Control Probability (TCP) in FLASH Radiotherapy? A Model-Based Analysis

**DOI:** 10.3390/ijms24065118

**Published:** 2023-03-07

**Authors:** Hans Liew, Stewart Mein, Thomas Tessonnier, Amir Abdollahi, Jürgen Debus, Ivana Dokic, Andrea Mairani

**Affiliations:** 1 Clinical Cooperation Unit Translational Radiation Oncology, German Cancer Consortium (DKTK) Core-Center Heidelberg, National Center for Tumor Diseases (NCT), Heidelberg University Hospital (UKHD) and German Cancer Research Center (DKFZ), 69120 Heidelberg, Germany; 2 Division of Molecular and Translational Radiation Oncology, Heidelberg Faculty of Medicine (MFHD) and Heidelberg University Hospital (UKHD), Heidelberg Ion-Beam Therapy Center (HIT), 69120 Heidelberg, Germany; 3 Heidelberg Institute of Radiation Oncology (HIRO), National Center for Radiation Oncology (NCRO), Heidelberg University Hospital and German Cancer Research Center (DKFZ), 69120 Heidelberg, Germany; 4 Department of Radiation Oncology, University of Pennsylvania, Philadelphia, PA 19104-6303, USA; 5 Heidelberg Ion-Beam Therapy Center (HIT), Department of Radiation Oncology, Heidelberg University Hospital, 69120 Heidelberg, Germany; 6 Department of Radiation Oncology, Heidelberg Institute of Radiation Oncology (HIRO), University Hospital Heidelberg, National Center for Tumor Diseases (NCT), 69120 Heidelberg, Germany; 7 Clinical Cooperation Unit Radiation Oncology, German Cancer Consortium (DKTK) Core-Center Heidelberg, National Center for Tumor Diseases (NCT), Heidelberg University Hospital (UKHD) and German Cancer Research Center (DKFZ), 69120 Heidelberg, Germany; 8 Medical Physics Unit, National Centre of Oncological Hadrontherapy (CNAO), 27100 Pavia, Italy

**Keywords:** ionizing radiation, FLASH, UNIVERSE, dose-rate, DNA repair, modeling, tumor volume, TCP

## Abstract

Reports of concurrent sparing of normal tissue and iso-effective treatment of tumors at ultra-high dose-rates (uHDR) have fueled the growing field of FLASH radiotherapy. However, iso-effectiveness in tumors is often deduced from the absence of a significant difference in their growth kinetics. In a model-based analysis, we investigate the meaningfulness of these indications for the clinical treatment outcome. The predictions of a previously benchmarked model of uHDR sparing in the “UNIfied and VERSatile bio response Engine” (UNIVERSE) are combined with existing models of tumor volume kinetics as well as tumor control probability (TCP) and compared to experimental data. The potential TCP of FLASH radiotherapy is investigated by varying the assumed dose-rate, fractionation schemes and oxygen concentration in the target. The developed framework describes the reported tumor growth kinetics appropriately, indicating that sparing effects could be present in the tumor but might be too small to be detected with the number of animals used. The TCP predictions show the possibility of substantial loss of treatment efficacy for FLASH radiotherapy depending on several variables, including the fractionation scheme, oxygen level, and DNA repair kinetics. The possible loss of TCP should be seriously considered when assessing the clinical viability of FLASH treatments.

## 1. Introduction

The possible emergence of the FLASH effect—the sparing of normal tissue while maintaining tumor control—after irradiations at dose-rates exceeding several tens of Gy per second, has recently spurred a surge of studies attempting to characterize and rationalize the phenomenon. With the sparing of biological systems at ultra-high dose-rates (uHDR) being consistently described and discussed since the late 1960s [1,2,3], it was the reports of an absence of such sparing effects in tumors emerging in the mid-2010s that marked the birth of the idea of “FLASH radiotherapy”, nurturing hopes of establishing an exploitable mechanism to increase the 55therapeutic window [4,5,6,7,8]. Already at an early stage it was recognized that the uHDR sparing effect was dependent on the oxygen level within the target [1,9,10,11] and more recent studies appear to confirm this [12,13]. Furthermore, the survival curves of cells after irradiation with uHDR were described to mimic the behavior of those obtained under hypoxia beyond a given breaking point dose (~10 Gy) in early studies [1,9,10]. These observations have been generally understood as indicators that the underlying mechanism needs to involve oxygen in a central role, inspiring the suggestion of radical-radical interactions [2,14,15]—and most prominently—radiative oxygen depletion [1,6,7,16,17,18] as a possible driver of the sparing effect. On the other hand, it remains unclear and actively debated why a sparing effect is observable in normal tissue but appears to be absent in tumors [7,19,20].

Experimentally, sparing of normal tissues has been shown using an array of different endpoints and assays, including neural functions, lung fibrosis, and skin toxicity, while the—for the FLASH effect so central—absence of sparing in tumors is predominantly deduced from the observation of non-significant differences between in vivo tumor growth kinetics [8]. For example, in the recent studies of Diffenderfer et al. and Montay-Gruel et al., [21,22] the tumor volumes showed no significant difference throughout 30–50 days after irradiation with uHDR and standard dose-rates (SDR) for different applied doses and fractionation schemes. Such findings are often implicitly extrapolated to suggest the iso-effectiveness of tumor treatment by uHDR beam in clinical situations. However, clinical success of a treatment regime is mostly quantified using measures such as the tumor control probability (TCP), with no trivial connection to tumor growth kinetics. Should FLASH radiotherapy lead to a reduction of TCP, further dose-escalation might be necessary to avoid undertreatment of the tumor, increasing however the burden on NT. This might limit the expected increase in the therapeutic window in FLASH radiotherapy.

In the first half of this work, predictions of the surviving fraction of clonogens by the previously established “UNIfied and VERSatile bio response Engine” (UNIVERSE) are extended using existing descriptions of tumor volume kinetics and compared to data including different doses, dose rates and fractionation schemes, taken from the mentioned publications by Diffenderfer et al. and Montay-Gruel et al., to assess the degree of agreement. The UNIVERSE is a multipurpose mechanistic modeling framework of radiation action in biological systems based on the clustering of radiation induced DNA double strand breaks (DSB) in sub-domains of a cell nucleus [23,24,25,26,27,28]. In an earlier publication, DSB repair and oxygen depletion/re-oxygenation kinetics were included, enabling it to describe not only “classical” dose-rate effects based on the time-dependent processing of DSB, but also sparing phenomena at uHDR for sparsely ionizing radiation [25]. Recently, the framework was refined to enable the inclusion of the explicit temporal pulse structure of the irradiation and to consider oxygen dependent damage fixation kinetics in the sub-millisecond range [27]. The dose-rate dependent predictions of the model, including those of uHDR sparing effects, were shown to match a panel of in vitro and in vivo data acting as benchmarks [25,27].

For the second half of this work, the model is further developed to predict the tumor control probability (TCP) based on methods proposed by Shuryak et al. [29] and is benchmarked using clinical TCP data of non-small cell lung cancer (NSCLC) gathered by the same group. Consequently, the variation of TCP predicted by the created framework as a function of the applied dose-rate and its dependency on other factors such as the oxygen concentration and applied fractionation scheme is investigated. Ultimately, the potential implications of the predictions for the therapeutic application of uHDR beams are discussed.

## 2. Results

### 2.1. Tumor Growth

**Figure 1:**
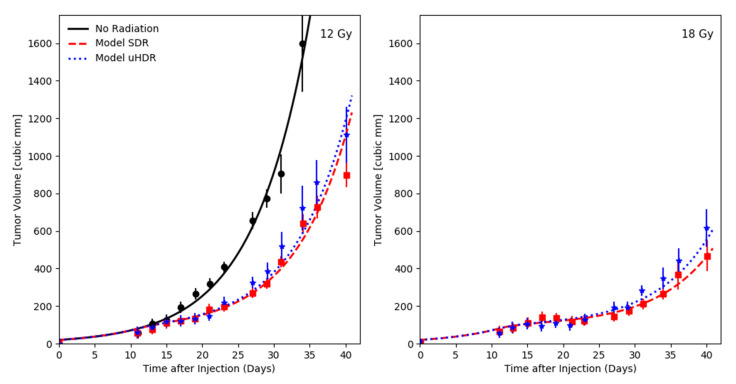
Growth kinetics of tumors in the flank of mice induced by injection of pancreatic tumor cells (MH641905) taken from Diffenderfer et al. [21] with respective UNIVERSE predictions. The tumors received either no radiation (black) or were irradiated 10 days after injection at the entrance of a 230 MeV proton beam, giving either 12 (**left**) or 18 Gy (**right**) at 63 Gy/s (uHDR, blue) or 0.74 Gy/s (SDR, red).

Diffenderfer and colleagues investigated the growth kinetics of subcutaneous pancreatic tumors (MH641905) [21], shown in Figure 1. The tumors were either untreated (No radiation) or irradiated 10 days after injection at the entrance of a 230 MeV proton beam, giving either 12 or 18 Gy at 63 Gy/s (uHDR) or 0.74 Gy/s (SDR). Based on the observed growth behavior of the non-irradiated tumors, an exponential growth model was chosen for this analysis (cp. Section 4). Within UNIVERSE, the kinetics of DNA damage repair are described by the exponential processing of isolated and clustered DSB, characterized by the half-life times $T_{iDSB}^{1/2}$ and $T_{cDSB}^{1/2}$, respectively [25]. For the predictions in this section, half-life times of $T_{iDSB}^{1/2}$ = 20 min and $T_{cDSB}^{1/2}$ = 5 h [30], as well as an oxygen concentration of 1% [31] were chosen based on the literature values. To enable comparability to other possible approaches of describing radiation action, the radiosensitivity of the tumors were described using the linear quadratic model (LQM) and its common parameters α and β (cp. Section 4). As only single fractions at two dose levels were available in this data set, the α/β-ratio was set to 10 Gy, as a typical value for tumors. Cells which do not produce a colony of the size necessary to be labeled as “survived” in a clonogenic assay can still produce a limited amount of off-spring which can contribute to the growth of the tumor. Thus, the halting of the growth of inactivated clonogens was delayed by a timestep sampled from an exponential distribution with the half-life time $T_{d}^{1/2}$ (cp. Materials and Methods). The α and $T_{d}^{1/2}$ values were optimized based on the R^2^ (coefficient of determination) of the SDR data, computed using the logarithmic volume. The optimum R^2^ of the SDR data set was found to be an average of 0.96 and yielded an average R^2^ for the uHDR dataset of 0.96 at an α value of 0.045 Gy^−1^ and a $T_{d}^{1/2}$ of 1.4 days.

**Figure 2:**
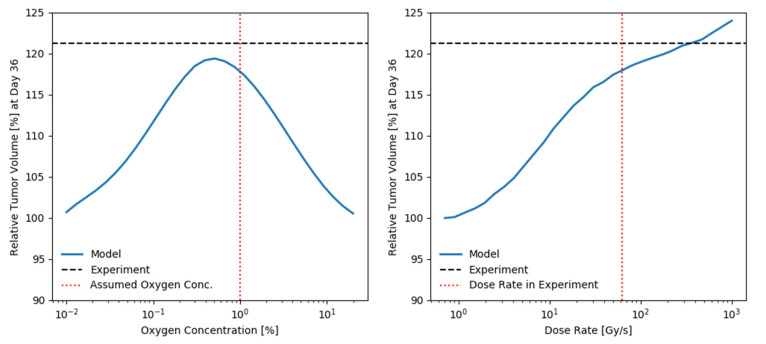
Predicted relative tumor volume 36 days after irradiation with 18 Gy between SDR and uHDR applying model parameters obtained for the data by Diffenderfer et al. [21] in dependency of the assumed oxygen level and dose-rate of the uHDR beam. For reference, the value measured in the original study is shown as a horizontal black dashed line. The oxygen level assumed for the predictions in Figure 1 and the dose rate applied in the experiment are marked by a vertical red dotted lines in the respective panels. (**left**): For the applied parameters, a maximum sparing is seen at an oxygen concentration of about 0.5% and the tumor volume of the uHDR group is predicted to be increased by 10% or more for any oxygen concentration between ~0.07 and 4%. (**right**): The relative tumor volume is seen to continuously increase with the dose-rate of the uHDR radiation.

In order to assess the impact of the assumed oxygen level and dose-rate as well as the sensitivity of the prediction to these parameters, the predicted relative tumor volume 36 days after irradiation with 18 Gy between SDR and uHDR is shown in Figure 2 in dependency of the assumed oxygen level and dose-rate of the uHDR irradiation, respectively. The measured value of the relative tumor volume is shown as a horizontal black dashed line for reference. The oxygen level assumed for the predictions and the dose rate applied in the experiment are marked by a vertical red dotted line. The relative tumor volume is seen to continuously increase with the dose-rate of the uHDR radiation (left panel Figure 2). A maximum of relative tumor volume is seen at an oxygen concentration of 0.5% and the tumor volume of the uHDR group is predicted to be increased by 10% or more for any oxygen concentration between ~0.07 and 4%.

**Figure 3:**
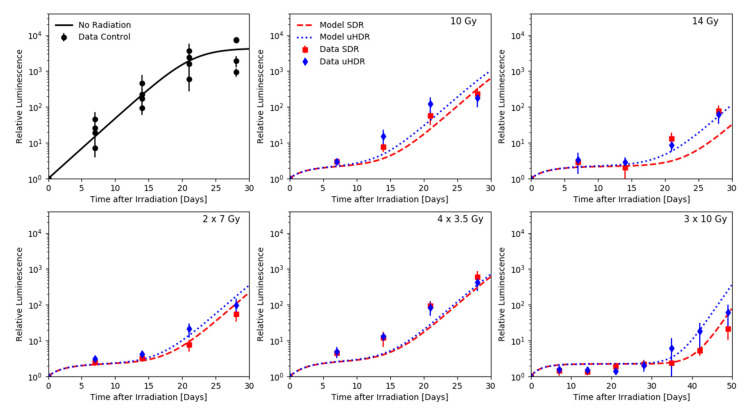
Growth of orthotopic glioblastoma based on H454 cells implanted in the striatum of nude mice, receiving no radiation (black) or irradiated 3 days post-injection with a 6 MeV electron beam set to either 0.1 Gy/s (SDR, red) or a single 1.8 µs pulse (uHDR, blue) obtained via bioluminescence imaging by Montay-Gruel et al. [22] with respective UNIVERSE predictions. Both dose rates were applied following different fractionation schemes: single fractions of 10 and 14 Gy, two fractions of 7 Gy, four fractions of 3.5 Gy as well as three fractions of 10 Gy.

In their 2021 study, Montay-Gruel and his colleagues measured the growth of orthotopic glioblastoma based on H454 cells implanted in the striatum of nude mice [22], which were irradiated 3 days post-injection with a 6 MeV electron beam set to either 0.1 Gy/s (SDR) or a single 1.8 µs pulse (uHDR). The development of the tumor size was measured via bioluminescence imaging. In this study, the relative increase in the luminescence intensity was assumed to approximate the relative tumor volume to a sufficient degree. Both modes of irradiation were administered using various fractionation schemes: single fractions of 10 and 14 Gy, two fractions of 7 Gy, four fractions of 3.5 Gy as well as three fractions of 10 Gy (Figure 3). The reported measurements of the growth kinetics in the non-irradiated control group were pooled together (top left panel Figure 3) and based on their trend the logistic growth model was chosen for this data set. Again, an oxygen concentration of 1% [31] and DNA damage repair half-life times of $T_{iDSB}^{1/2}$ = 20 min and $T_{cDSB}^{1/2}$ = 5 h [30] were chosen for the fast and slow component, respectively, based on the literature values. The value of the α/β-ratio, α and $T_{d}^{1/2}$ were optimized based on the R^2^ (coefficient of determination) of the SDR data, computed using the logarithmic volume. The optimum R^2^ of the SDR data set was found to be an average of 0.83 and yielded an average R^2^ for the uHDR dataset of 0.88 at an α/β-ratio of 12 Gy, an α value of 0.28 Gy^−1^ and a $T_{d}^{1/2}$ of 1.0 days.

### 2.2. Clinical Tumor Control Probability Analysis

**Figure 4:**
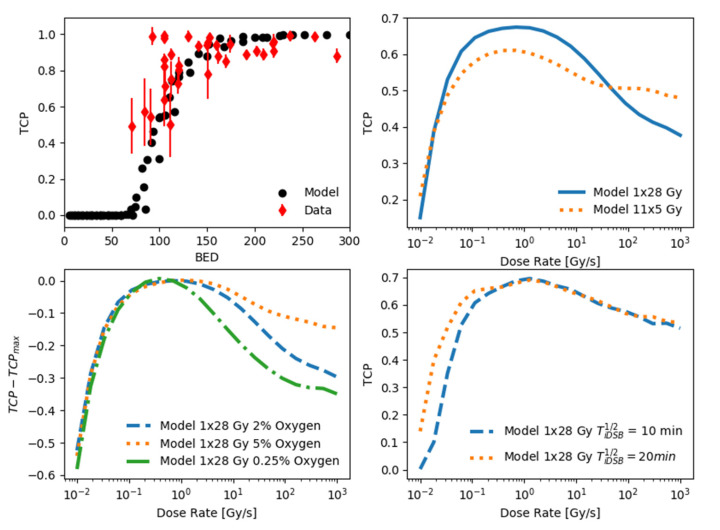
Potential reduction of tumor control probability (TCP) at uHDR. (**top left**): TCP data based on 2028 non-small cell lung cancer (NSCLC) patients (black circles) treated with different fractionations of stereotactic radiotherapy as a function of biologically equivalent dose (BED) compiled by Shuryak et al. [29] (red diamonds) with UNIVERSE prediction. (**top right**): UNIVERSE predictions of TCP as a function of the dose-rate for two fractionation schemes with comparable effect at conventional dose-rates (one fraction of 28 Gy, blue line; eleven fractions of 5 Gy, dotted orange line). Both approaches exhibit a considerable loss of TCP towards uHDR, with a larger reduction predicted for the single fraction approach. (**bottom left**): The predicted decline of TCP from its maximum value is dependent on the assumed oxygen level. Within the range of typical values of oxygen concentrations in tumors (0.25% shown as green dash-dotted line to 5% shown as orange dotted line [32,33]), the potential loss increases towards lower oxygen concentrations. (**bottom right**): Observation of a sparing effect can also be influenced by the effect at the conventional reference dose rate. Depending on the assumed DNA repair half-life times ($T_{iDSB}^{1/2}$) and the reference dose-rate iso-effectiveness or even increased effectiveness at uHDR could be predicted.

In 2015, Shuryak et al. published a study in which they collected a large number of tumor control probability data sets for patients receiving stereotactic radiotherapy using different fractionation schemes, including 2028 patients treated for non-small cell lung cancer (NSCLC) [29], shown in the top left panel of Figure 4 as a function of the biologically equivalent dose (BED). To model the tumor control probability in UNIVERSE, the approach proposed by Shuryak et al., which supposes a heterogeneous distribution of radiosensitivity values within the tumor, was adapted for use in our framework (cp. Materials and Methods). Following the suggestions by Shuryak et al., the shape parameter *g* of the Gamma distribution describing the radiosensitivity is set to 5, the number of clonogens within the tumor *N* is set to 10^5^, the expected value of α  is set to 0.4 Gy^−1^, and the α/β-ratio is set to 10 Gy. Furthermore, an oxygen level of 2% for NSCLC [32] and DNA damage repair half-life times of $T_{iDSB}^{1/2}$= 20 min and $T_{cDSB}^{1/2}$ = 5 h [30] were chosen for the fast and slow component, respectively, based on the literature values. To assess the possible impact of increased dose-rates for different fractionation schemes, the simulated TCP for two fractionation schemes with comparable effect at conventional dose-rates are shown over the dose-rate in the top right panel of Figure 4. After an initial increase in the TCP with the dose rate, both fractionation schemes start to exhibit a considerable loss of TCP at dose rates above a couple of Gy/s. While the TCP of the single fraction continues to decrease with the dose rate, at a given point its effectiveness falls below that of the fractionated irradiation which appears to reach a plateau. However, both fractionation schemes show a second phase of TCP reduction towards the highest dose rates analyzed in this study. To investigate the potential influence of the oxygenation on the predicted loss of TCP towards uHDR, the predictions for the single fraction of 28 Gy were re-calculated assuming the radiosensitivity parameters given by Shuryak et al. to be obtained under 5% and 0.25% (bottom left panel Figure 4). These two values are supposed to represent the upper and lower end of oxygen observed in common tumors [32,33]. The highest potential loss of TCP in comparison to its maximum over the dose rate ($TCP_{max}$) was observed for the most hypoxic setting (~35 percentage points), while the lowest was seen for the least hypoxic setting (~10 percentage points). Decreasing the assumed repair half-life time of the fast component of DNA damage repair ($T_{iDSB}^{1/2}$) from 20 to 10 min results in a reduction of TCP for dose-rates below ~1 Gy/s (bottom right panel Figure 4) in an analysis of the TCP over the dose-rate assuming an oxygen concentration of 5%.

## 3. Discussion

Using a basic exponential model of tumor volume growth and model parameters derived solely from the data obtained under SDR, our framework was able to appropriately predict the growth kinetics of tumors treated with uHDR as reported by Diffenderfer et al. [21] (Figure 1). The model reproduces the slight difference in the mean tumor volume over time, especially visible in the high dose setting (right panel Figure 1). The predicted bell-shaped dependence of the relative tumor volume as a function of the oxygen concentration (left panel Figure 2) can be explained as follows: at very low oxygen concentrations, there is essentially no oxygen to be depleted by the beam, thus the change in radiosensitivity is minimal. On the other hand, at high oxygen concentrations, the depleted amount of oxygen is too small in comparison to that available in the environment to substantially shift the radiosensitivity of the system. While the model slightly underestimates the measured difference in tumor volumes after 36 days, it is noteworthy that for a range of oxygen concentrations spanning nearly two orders of magnitude (~0.07% to ~4%) it predicts a volume difference of above 10%. A substantial sparing could have thus been expected even if the targets oxygenation would vary within the common range of tumors [32,33]. The relative tumor volume is found to increase monotonically as a function of the dose rate (right panel Figure 2). Interestingly, given the assumed parameters, a volume difference of above 10% is predicted to arise around the order of tens of Gy/s, coinciding with the dose-rate threshold at which sparing effects have repeatedly been reported to arise [7].

The data obtained by Montay-Gruel et al. [22] not only allowed to benchmark our framework at additional doses and dose rates but also under consideration of various fractionation schemes. In order to analyze the dataset, a common but more complex logistic function was used to describe the tumor volume kinetics (Figure 3). While the model parameters were again derived from the SDR data only, the frameworks prediction of the absolute values are not as accurate as those for the dataset by Diffenderfer et al. However, one might need to consider that the data itself could be subject to a larger intrinsic variability as indicated by the measurements of the control group (top left panel Figure 3). Furthermore, the model reproduces the differences between the tumor volumes as well as its trends satisfactorily, with the sparing effects generally increasing with the dose applied per faction (Figure 3). The dataset acquired after irradiation with 14 Gy (top right panel Figure 3) appears to not follow this trend. Here, the model predicts a clear differentiation between the tumor volumes after SDR and uHDR irradiation that is not visible in the data, although this is the highest single dose delivered in this analysis. At the same time, it is noteworthy that, within the data taken from Montay-Gruel et al. for this work, this single 14 Gy fraction and 4 fractions of 3.5 Gy where the only settings which showed no normal tissue sparing in the original study.

The analysis of tumor volume kinetics data has shown that UNIVERSE is generally able to assess and predict this key in vivo endpoint using simple and established models of tumor growth based on the inactivation of clonogens. Analogously, an approach suggested by Shuryak and his colleagues [29] was applied on survival fractions computed by UNIVERSE to predict the clinically relevant tumor control probability (TCP). Their approach essentially introduced a distribution of radiosensitivity within the population of clonogens that make up the tumor. Following this method, the clinical TCP for NSCLC patient treated with different fractionation schemes of stereotactic radiotherapy could be reproduced as a function of the biologically effective dose (BED) [34] (top left panel Figure 4). The persisting variance of the predictions did not allow a meaningful calculation of a measure for the goodness of fit, however the predictions appear to describe the data reasonably well. The analysis shown in the top right panel of Figure 4 exemplifies how two fractionation schemes that are approximately iso-effective in the regimen of conventional dose rates could result in distinct tumor control at uHDR according to the presented model: while the predicted TCP of the fractionated approach with lower dose per fraction is shown to reduce by about 10 percentage points from about 60% to 50%, the high-dose single-fraction approach could experience a steeper drop of about 25 percentage points from about 65% to approximately 40% at the highest dose rates studied. Assuming iso-effectiveness in the tumor while transitioning to a high-dose and high dose-rate combination applied in FLASH radiotherapy could thus result in serious undertreatment of the tumor according to the model with the given set of parameters. Within the framework, this issue was even more pronounced when the assumed oxygen was decreased to a value typical for severely hypoxic tumors (0.25%), such as pancreatic cancers (bottom left panel Figure 4). For the single 28 Gy fraction, the potential loss of TCP grew to about 35 percentage points, while it shrank to about 10 percentage points if one assumed a value typical for well oxygenated tumors (5%), such as rectal carcinomas [32,33]. According to the model, the risk of undertreatment should thus be especially considered when the tumor is known or suspected to be at the lower end of typical oxygen levels or contains regions for which this holds true. On the other hand, one needs to keep in mind that the iso-effectiveness also involves the effect of the SDR radiation. As an example, let us assume that the single 28 Gy fraction of the TCP analysis is applied to a tumor under 5% oxygen with the dose rates used in the tumor growth kinetic study by Diffenderfer et al. (bottom right panel Figure 4): the SDR (0.74 Gy/s) would yield a TCP close to the predicted maximum of 70%, while the uHDR (63 Gy/s) would lead to a somewhat reduced value around 60%. However, if the SDR would be that of the study by Montay-Gruel et al. (0.1 Gy/s), its TCP would decrease due to the increased repair of DNA damages during the prolonged irradiation time. Assuming a reduced half-life time of the fast component of DNA damage repair of 10 min could even lead to iso-effectiveness between SDR and uHDR. Within this framework, it is thus possible that even if sparing effects are triggered, depending on the applied SDR and the swiftness of the DNA repair within the system, iso-effectiveness or even increased TCP is observed for the uHDR beam in comparison to its reference [35].

It is important to emphasize that this study makes no statement on the sparing effect in normal tissue or its potentially larger extent in comparison to that in tumors. While the possible reason behind the latter is still actively debated [7,19], mechanisms such as heterogeneous spatial oxygen distributions [20] or differences in the amount of oxygen depleted [36] could be implemented in our framework in a relatively straight-forward fashion. However, in contrast to endpoints such as the tumor control probability or tumor volume kinetics which have established connections to the survival of clonogens, these are lacking for complications associated with normal tissues that are thought to be organized in more hierarchical structures dependent on the tissue type [37]. The specific architecture of a normal tissue would not only be crucial for the description of radiation effects but could potentially lead to a differential response to uHDR beams in comparison to tumors. Furthermore, the predictions concerning the TCP presented in this study are supposed to be paradigmatic. The exact extent of the sparing effect is dependent on the parametrization of the oxygen effect and the description of the oxygen depletion process as well as the chosen values of their variables. These parameters are probably not only subject to great variability throughout different tumor types and patients but could generally be different between in vitro, in vivo, and clinical situations.

However, the main intention of this study is to show that our model, with the same parameterization as previously benchmarked using experimental data [25,27] and capable of describing tumor growth kinetics data showing no statistically significant sparing at uHDR, would predict potentially substantial under-treatment by FLASH radiotherapy when applied to a clinical TCP dataset. Even if the reduction of TCP is in fact lower than predicted in this example, it shows that the absence of statistically significant differences in tumor growth kinetics might not necessarily ensure “iso-effectiveness” for clinical endpoints. One could argue that despite a loss in therapeutical efficacy, normal tissue sparing might still be larger, ultimately increasing the therapeutic window. Yet, in a recent comprehensive study of available in vivo data on normal tissue sparing, Böhlen et al. [38] found that the average iso-effective ratio of doses applied at SDR and uHDR were “0.95 ± 0.11 for all data below 10 Gy, 0.92 ± 0.06 for mouse gut data between 10–25 Gy, and 0.96 ± 0.07 and 0.71 ± 0.06 for mammalian skin reaction data between 10–25 Gy and >25 Gy, respectively”. In a separate study, Böhlen et al. [39] argued, that these values would not suffice to regain the amount of sparing of late responding tissues lost by switching to hypofractionated treatments in most clinically relevant scenarios. Our study suggests that it is not far-fetched that similar values could be observed for the TCP, which would further limit the clinical viability of FLASH radiotherapy. While clinical trials involving FLASH radiotherapy still face a variety of challenges [40] and we are aware of regulatory hurdles, in vivo studies of tumor growth kinetics should try to include a larger number of specimens to improve the sensitivity for the potentially small differences in growth that may evolve to a more impactful change in tumor control. Ultimately, the question posed in the title will have to be answered via direct measurements of the TCP, which a few in vivo studies have presented with promising results for FLASH radiotherapy [41,42]. However, in the lights of the number of possible variables showcased in this study as well as the conclusions by Böhlen et al. mentioned above, we again see the necessity for more experiments with larger amounts of measurements to ensure sparing of the TCP can be excluded in all relevant cases, as even small amount of sparing might diminish the potential of FLASH radiotherapy. While the sparing of normal tissue has been at the center of attention in most studies, we should recall—given the background of its experimental evidence for uHDR sparing reaching back to the early 1960s—that the non-sparing of tumors is the defining aspect of the FLASH effect.

## 4. Materials and Methods

### 4.1. UNIVERSE

The surviving fraction of clonogens are predicted using the “UNIfied and VERSatile bio response Engine” (UNIVERSE). It was developed to serve as a framework for the mechanistic modeling of radiation action in biological systems and its numerous modifiers. It has already allowed to describe and predict the effect of DNA damage repair inhibition and kinetics for various radiation qualities [23,25,26,28], as well as the action of radical scavengers for sparsely ionizing radiation at different oxygenation levels [24]. At its core, the framework bases its prediction of the biological effect on the induction, clustering, and processing of DSB within nuclear domains on the micron scale. For this work, calculations are provided primarily by a previously established module developed to predict uHDR sparing effects based on oxygen depletion/re-oxygenation kinetics [25] which was extended by an explicit consideration of the temporal pulse structure and the oxygen dependent sub-millisecond damage fixation kinetics [27]. The reader is kindly referred to the latter two publications [25,27] for details on the methodology of these models.

### 4.2. Tumor Growth Model

The growth of untreated tumors can often be described with relatively simple ordinary differential equations [43]. In this study, either the exponential model Equation (1) or the logistic model Equation (2) is applied, depending on the tumor growth behavior observed in the control group of the datasets. The exponential model can be written as:

(1) $$V\left(t\right)=V\left(0\right)\cdot \exp\left(k_{g}\left(t-t^{\prime }\right)\right)$$

where *V*(*t*) is the tumor volume over time, $k_{g}$ is a growth constant and *t’* a constant shift in time.

In the logistic model the growth rate decreases as the volume reaches a saturation value $V_{max}$ [43] and its solution can be written as [44]:

(2) $$V\left(t\right)=V_{max}\;\cdot \;\frac{1}{1+\frac{V_{max}-V\left(0\right)}{V\left(0\right)}\;\exp\left(-k_{g}t\;\right)}$$

The model parameters are determined by fitting the equations to the tumor volume measurements of the control datasets, using the curvefit function of the scipy.optimize library in Python.

In order to model the impact of irradiation on the development of the tumor volumes, an approach introduced by Chvetsov et al. [45] and simplified by Wang and Feng [46] was adapted, where the volume of the tumor is assumed to be directly proportional to the number of cells within. In our implementation, the tumor volume is divided into sub-volumes, which are increased continuously using the chosen growth model of a given data set and the parameters derived from the control measurements. After each irradiation fraction, a fraction (1-S) of the sub-volumes growing at the time of irradiation are inactivated, representing the fraction of cells within the tumor volume that did not survive the irradiation. The survival fraction S is determined using the UNIVERSE framework, based on the parameters given for each setup. However, as cell survival is based on the ability of cells to form colonies of a given size, the sub-volumes that were inactivated were thought to continue growing for a given time before they ultimately stopped growing. The delay is sampled from an exponential distribution with a given half-life time $T_{d}^{1/2}$.

### 4.3. Tumor Control Probability Model

To predict the tumor control probability (TCP) of a given irradiation, we modified an approach proposed by Shuryak et al. [29] which assumes a heterogeneous distribution of radiosensitivity within the cells that make up a the tumor. In their version, the radiosensitivity is based on the linear quadratic model (LQM). The survival fraction of cells within the LQM after the irradiation with a dose *D* is given by:

(3) $$S=\exp\left[-\alpha D-\beta D^{2}\right]$$

where α and β are endpoint dependent radiosensitivity parameters. Given a fixed α/β-ratio, the heterogeneity of the radiosensitivity within a population is modeled by assuming that the α values follow a Gamma distribution, where the probability density function is given by:

(4) $$P\left(\alpha \right)=\alpha^{g}\;\cdot \exp\left[-\left(g+1\right)\;\alpha /<\alpha >\right]\;{\left(g+1\right)}^{\left(g+1\right)}/\left({<\alpha >}^{\left(g+1\right)}g!\right)$$

The parameter *g* determines the shape of the distribution while <α> describes the mean value of α. In their study, Shuryak et al. were able to show that this approach was also appropriate for high doses per fraction. Based on the P(α) derived by them to describe data gathered for conventional radiotherapy of NSCLC (cp. Figure 4 top left) and a given oxygen level (cp. Section 2 for exact parameters) the corresponding distribution of so-called lethality parameters can be derived. These describe the intrinsic sensitivity of cells to certain types of DNA damages in UNIVERSE. Based on the dose-rate independent lethality parameters and the number of damages simulated by UNIVERSE the survival fraction can then be predicted for any given dose-rate. If a fractionation scheme is applied, the survival values for each iteration simulated by UNIVERSE are first potentiated with the number of fractions before being averaged to yield the expectancy value of the survival within the heterogeneous population <S>. Using Poisson statistics, the probability that within a population of N cells no cell survives the irradiation and the tumor is controlled can be given by [37]:

(5) TCP=exp[−N·<S>]
